# Wide-field optical coherence tomography based microangiography for retinal imaging

**DOI:** 10.1038/srep22017

**Published:** 2016-02-25

**Authors:** Qinqin Zhang, Cecilia S. Lee, Jennifer Chao, Chieh-Li Chen, Thomas Zhang, Utkarsh Sharma, Anqi Zhang, Jin Liu, Kasra Rezaei, Kathryn L. Pepple, Richard Munsen, James Kinyoun, Murray Johnstone, Russell N. Van Gelder, Ruikang K. Wang

**Affiliations:** 1 University of Washington, Department of Bioengineering, 3720 15th Ave NE, Seattle, WA 98195, USA; 2 University of Washington, Department of Ophthalmology, 325 Ninth Avenue, Seattle, WA 98104, USA; 3 Carl Zeiss Meditec, Inc., Dublin, CA 94568, USA

## Abstract

Optical coherence tomography angiography (OCTA) allows for the evaluation of functional retinal vascular networks without a need for contrast dyes. For sophisticated monitoring and diagnosis of retinal diseases, OCTA capable of providing wide-field and high definition images of retinal vasculature in a single image is desirable. We report OCTA with motion tracking through an auxiliary real-time line scan ophthalmoscope that is clinically feasible to image functional retinal vasculature in patients, with a coverage of more than 60 degrees of retina while still maintaining high definition and resolution. We demonstrate six illustrative cases with unprecedented details of vascular involvement in retinal diseases. In each case, OCTA yields images of the normal and diseased microvasculature at all levels of the retina, with higher resolution than observed with fluorescein angiography. Wide-field OCTA technology will be an important next step in augmenting the utility of OCT technology in clinical practice.

Optical coherence tomography (OCT) is a non-invasive imaging modality that allows for the evaluation of the vitreous cavity, retinal layers, retinal pigment epithelium, and choroid. Since its initial description in 1991^1^, OCT technology has rapidly evolved with better sensitivity, increased depth of penetration, and higher resolution for imaging changes seen in retinal diseases. Fourier domain or wide-field OCT are only a few examples of this improving technology^2^. OCT imaging now plays a critical role in clinical trials studying age-related macular degeneration (AMD)^3^, diabetic macular edema^4^,^5^, and glaucoma^6^.

Currently, Fourier domain OCT can achieve an axial resolution from 1 to 15 um^7^,^8^,^9^. In addition, “swept-source” OCT has increased the field of view dramatically due to its speed and sensitivity advantages over its spectral domain counterpart. However, even with its ultra high-resolution, wide-field OCT is limited in its ability to provide information on the function of retinal structures. Fluorescein angiography (FA) and indocyanine green angiography (ICGA) have been the mainstays for evaluating functional blood flow involved in various retinal/choroidal diseases^10^,^11^. Ultra-wide-field fluorescein angiography (UWFA) systems now allow for visualization of functional vessels in an area up to 200° of retina^12^,^13^. The coverage of near twice as much retina compared to conventional fundus photographs and FA has been beneficial in many studies^14^,^15^,^16^ and is likely to alter the management of retinal diseases in future^13^. However, the invasive nature, time, and personnel requirements make traditional and wide-field FAs less desirable and less feasible, especially in busy clinical settings.

Recently, the introduction of OCT-based angiography (OCTA) has shown promising results in allowing non-invasive, functional imaging of retinal and choroidal vasculature^17^. However, the main challenges in OCTA include the limited field of view and eye motion artifacts^18^,^19^. The wide-field OCTA improves the narrow field of view in traditional OCTA, but some technical difficulties still exist. The constant, involuntary micro saccades during image acquisition remain the main obstacles to producing reliable, reproducible, wide-field imaging.

OCT-based microangiography (OMAG) is one of the leading technologies in OCT-based angiography^20^,^21^. This imaging modality has been used to evaluate retinal microvasculature and its associated pathologies previously^22^,^23^. Different from other current OCTA algorithms, for example decorrelation^24^, speckle variance^25^, and split-spectrum amplitude decorrelation (SSADA)^26^, OMAG uses both amplitude and phase in the OCT signals to extract the blood flow information within tissue volume. Adding a motion tracking system through an auxiliary real time line scan ophthalmoscope (LSO)^27^ in OMAG allows high-resolution, wide field imaging, which is an important next step in augmenting the utility of OCTA in clinical practice. In this paper, we present the first results of wide-field OMAG in six cases with no ocular pathology, non-proliferative diabetic retinopathy (NPDR), proliferative diabetic retinopathy (PDR), branch retinal vein occlusion (BRVO), retinitis pigmentosa (RP), and polypoidal choroidal vasculopathy (PCV). Additionally, we describe the comparability of wide-field OMAG results to FA or ICGA.

## Methods

Clinical and imaging data were collected prospectively from patients attending the retina clinics at the University of Washington, Seattle, WA between January 2014 and February 2015. This study was approved by the Institutional Review Board of the University of Washington and informed consent was obtained from all subjects. This study followed the tenets of the Declaration of Helsinki and was conducted in compliance with the Health Insurance Portability and Accountability Act.

All patients underwent imaging with a 68 kHz Cirrus-5000 SD-OCT based angiography prototype (Carl Zeiss Meditec Inc., USA) that operates at a central wavelength of 840 nm. To achieve OMAG imaging of retinal vasculature, a repeated B-mode scanning protocol was implemented in the prototype [Supplementary Figure 1]. A number of repeated B-scans were acquired at each position over the slow axis direction (y-axis). For each B-scan, 245 A-lines were sampled in a lateral distance along the x-axis (fast axis direction) and a total of 245 B-scans were obtained along the y-axis (slow axis direction). The cluster scan was defined as the number of repeated B-scans at the same location; hence each position in the slow axis represented a cluster scan. Four repeated B-scans were performed in the current study to extract the blood flow signal. The time interval between two successive B-scans was 3.7 ms, which corresponds to a B-scan acquisition rate of 270 frames per second. The total time for a single volume acquisition was about 3.6 seconds, excluding the adjustment time before the data collection.

In this study, the Cirrus-5000 SD-OCT based angiography prototype was equipped with an additional and unique feature: motion tracking through an auxiliary real time line scan ophthalmoscope (LSO)^28^, which provides motion-free vascular imaging *in vivo* and subsequent montaged images that allow for an unprecedented, large field of view (FOV) using the OMAG technique. The montage scanning protocol guided the OCT probe beam to scan at a predefined grid [Supplementary Figure 2] where a single OMAG cube scan was performed at each grid one by one. Adjacent cubes had a 10% overlap for the purpose of later stitching among cubes to generate larger composite field of view. In the current study, minimum array for the grid was 3 × 3, giving a FOV of 6.8 × 6.8 mm^2^ while maximum array is 4 × 6, providing a coverage of 9.0 × 13.4 mm^2^. Considering the acquisition time and patient tolerance, different fields of view were applied to the patient scans. The OMAG algorithm^21^,^29^,^30^ was applied to all the volumetric datasets, and the large FOV en face OMAG was obtained by stitching the images automatically by use of a software coded with Matlab. With a desktop computer with Intel Core i7 processor, the entire data processing, including montaging, was ~36 min (with each cube taking ~4 min) for 3 × 3 array cubes.

A complex function based differentiation algorithm was applied to extract *in vivo* blood flow information as described previously^29^. In brief, both the amplitudes and phases were utilized to differentiate the OCT signals between repeated B-scans to contrast blood flow information. Before the algorithm was applied, displacements caused by involuntary eye movements among repeated B-scans were compensated by using two-dimensional cross correlation between two adjacent B-scan images^21^,^31^.

Retinal and choroidal layers were segmented using a semi-automated retinal layer segmentation algorithm^32^. Three main retinal layers were segmented in all patients: a superficial retinal layer (SRL) that includes the ganglion cell layer and the inner plexiform layer; a deep retinal layer (DRL) that includes the inner nuclear layer and the outer plexiform layer; an outer retinal layer (ORL) that includes the outer nuclear layer and the external limiting membrane. Of note, the internal limiting membrane/nerve fiber layer and the photoreceptor inner and outer segment layers are segmented separately and can be evaluated in pathologic eyes.

The choroid was segmented between the choriocapillaris and the deep choroid layers. The choriocapillaris was defined as a 30-micron slab beneath the retinal pigment epithelium (RPE) layer. The deep choroid was defined as the slab between the choriocapillaris and the sclera. This segmentation was performed using the OCT cross-sectional structural images based on the intensity differences between retinal layers and was applied to the entire three-dimensional dataset.

A software coded with Matlab was used to render, project and color the three dimensional structure of the retina and microvasculature. Segmentation allowed for the visualization of the microvasculature in different layers of the retina and choroid. The en face images were created by using a maximum amplitude projection (MAP) method within the layer of interest and then used to correlate the findings between OMAG and FA images. Before the MAP, a 3 × 3 × 3 pixel-kernel Gaussian filter was applied to enhance the quality of the images.

## Results

We have characterized the imaging capabilities of OMAG in evaluating the vitreous cavity, retinal layers, RPE, and choriocapillaris. The following disease processes represent retinal pathologies within different layers of tissues and demonstrate the clinical usefulness of wide-field and high definition images obtained by OMAG with motion tracking LSO.

### Overview: Normal Retinal Microvasculature

**Figure 1:**
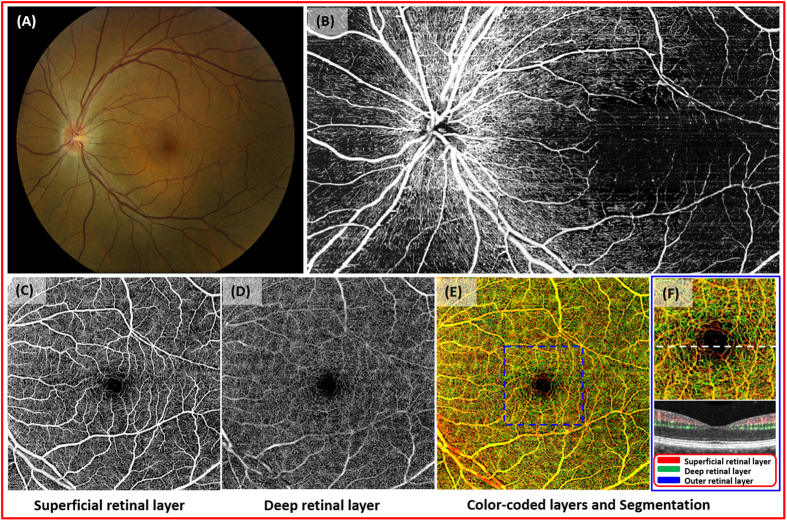
Optical coherence tomography based microangiography (OMAG) images of a 49 year-old Asian male. (**A**), fundus photograph of normal retina. (**B**), montaged OMAG images of the nerve fiber layer. Radial pericapillary network within the nerve fiber layer is noted. (**C**), the superficial retinal layer (SRL) slab contains the vascular network within the ganglion cell layer and the outer plexiform layer. The arcade vessels and the fine capillaries are shown. (**D**), the deep retinal layer (DRL) demonstrates deeper capillary network. (**E**), the whole retinal layer slab composed of the SRL, the DRL, and the outer retinal layer (ORL) allows visualization of the superficial, intermediate, and deep retinal capillary plexuses. Different colors identify various levels of the retina. (**F**), the magnified image of the central macula (identified as in the blue box from (**E**)). The cross-sectional flow image of the area marked with white dashed line on the magnified OMAG image. Blood flow detected in SRL, DRL, ORL in red, green, blue, respectively. No flow is appreciated in the ORL.

With motion tracking LSO, a 3 × 5 cube scan is implemented and motion-free, high-resolution images are obtained. This enables montaging of 15 scans to cover up to 60 degrees (6.8 × 11.2 mm) of retina in any selected layer (Fig. 1B). Intricate details of various pathologies can be viewed at one glance. The normal retinal segmentation scheme includes superficial, deep and outer layers. However, depending on the level of the pathology, other slabs are possible, including the nerve fiber layer and photoreceptor layer.

In a 49 year-old male with no significant ocular history (Fig. 1A), both dense capillary networks and larger retinal vessels are observed (Fig. 1B–F). A radial capillary network originating from the optic nerve can be seen in the nerve fiber layer (Fig. 1B). Large vessels with capillaries are located in the superficial retinal layer (ganglion cell and inner plexiform layers) while the deep retinal layer (inner nuclear and outer plexiform layers) contains denser capillary networks (Fig. 1 C,D). The outer retinal layer is devoid of vessels in normal patients, and this was confirmed with our images (not shown). The cross sectional image of OMAG demonstrates blood flow within superficial and deep middle retinal layers (Fig. 1F).

### Pre-retinal and Inner Retinal vasculature: Diabetic Retinopathy

The integrity of the retinal microvasculature is essential in defining different stages of diabetic retinopathy. At present, the severity of diabetic retinopathy is determined on clinical exam, assisted by stereoscopic fundus photos or FA^33^,^34^. However, recent studies demonstrate that OCT imaging may provide a more sensitive detection of proliferative diabetic retinopathy^35^,^36^. A correlation between retinal histopathologic findings of diabetic retinopathy and OCT images of intraretinal microvascular abnormalities and neovascular complexes has been discussed previously, implying that different stages of the proliferative disease processes may be monitored with precision in patients over time^37^. In clinical practice, non-invasive imaging of disease progression in real time may result in OCT-based findings that can be used in clinical studies to evaluate more effective means of early intervention and treatment.

**Figure 2:**
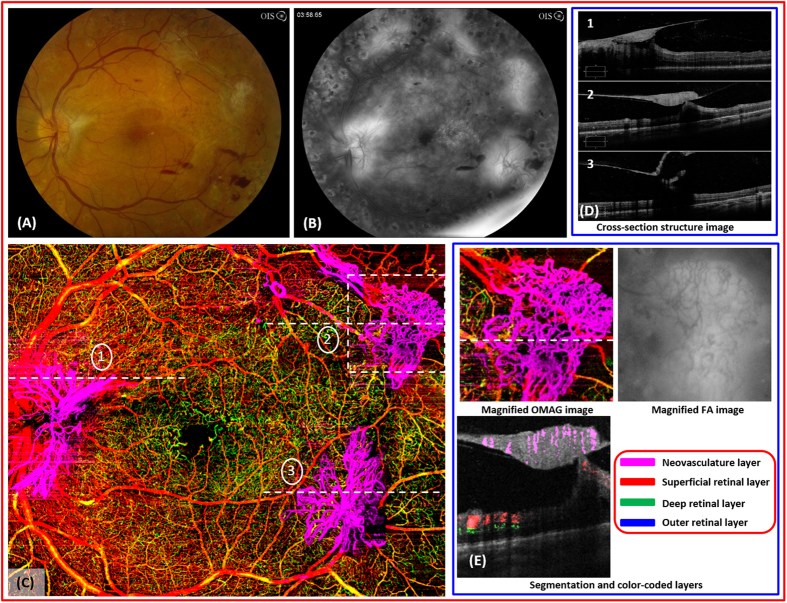
OMAG images of a 31 year-old male with proliferative diabetic retinopathy. (**A**), fundus photo of proliferative diabetic retinopathy in the left eye. There are multiple, large fibrovascular complexes associated with pre- and intraretinal hemorrhages. (**B**), the late frame of the fluorescein angiography demonstrates diffuse leakage from several areas of active neovascularization. (**C**), the OMAG image of the whole retinal layer shows three large neovascular complexes that have penetrated into the vitreous cavity. (**D**), the structural optical coherence tomography shows the disruption of internal limiting membrane by the neovascular complexes (dashed lines indicated with #1,2,3 in (**C**)) and their growth into the vitreous cavity. (**E**), high-definition details of the vascular complex such as the vessel caliber, volume, density of capillary network can be appreciated compared to the FA. The flow OMAG image shows the evidence of vascular flow within the superotemporal neovascularization of elsewhere marked with a white dashed box in (**C**).

A 31 year-old male with proliferative diabetic retinopathy of the left eye (Fig. 2A) and non-proliferative diabetic retinopathy of the right eye underwent a 4 × 5 single cube scan and 3 × 3 single cube scan, respectively. Striking images of neovascularization of the disc and elsewhere were detected easily by OMAG due to the location of the neovascular complexes and the high flow within the lesions (Fig. 2C,E). The structural cross-sectional OCT image is complementary to the findings on OMAG (Fig. 2D) and demonstrates the disruption of the internal limiting membrane. Active neovascularization of the disc and elsewhere are characterized by leakage on the FA, and the margins are blurred on the late phase (Fig. 2B). However, these lesions remain as well-delineated structures with visible vascular networks on OMAG, and further quantification of these structures is possible (Fig. 2E). In addition, the flow within these lesions is easily detectable with the flow image (Fig. 2E).

In addition to characterizing proliferative changes, OMAG can assess capillary perfusion in the macula. Assessing the degree of macular ischemia is critical in determining the visual prognosis of patients with diabetic retinopathy. The irregularity or the enlargement of the foveal avascular zone (FAZ) is characteristic of diabetic retinopathy and strongly correlated with the degree of capillary nonperfusion within the retina^38^. Thus, perfusion status of the FAZ is particularly important in assessing the likelihood of poor response to any therapy.

**Figure 3:**
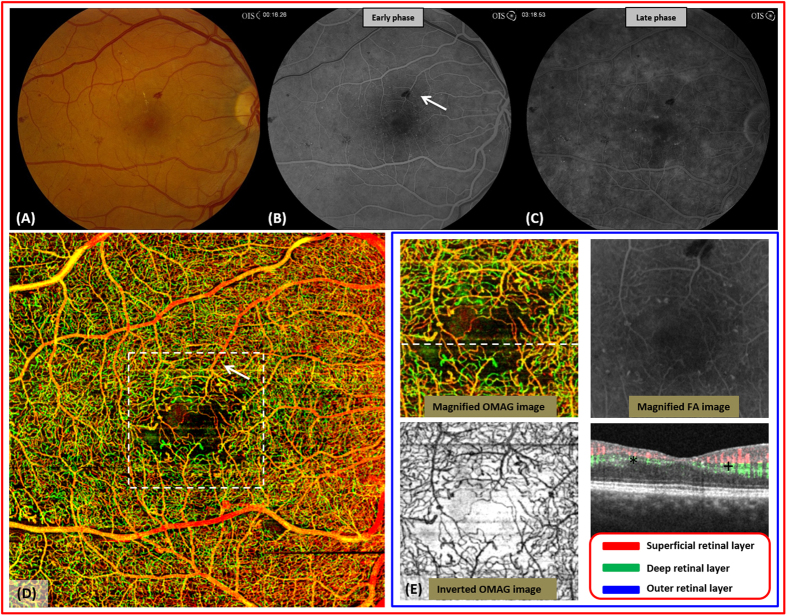
OMAG images of severe non-proliferative diabetic retinopathy in a 31 year-old male. (**A**), fundus photo of severe nonproliferative diabetic retinopathy in the right eye shows several intraretinal hemorrhages and microaneurysms (MA). (**B,C**), the early and late frames of the fluorescein angiography show diffuse late leakage from MA’s. (**D**), an enlarged and irregular foveal avascular zone (FAZ) is associated with several dilated vascular bulbs as shown on the whole retinal OMAG image. There is no blockage from hemorrhage on the OMAG scan (arrow). (**E**), the magnified OMAG image of central macula marked with white dashed box in (**D**). Microaneurysms identified in the inverted display of OMAG image (dark appearance) show excellent agreement with those identified in FA image. The flow image shows decreased flow in both superficial and deep layers in the nasal fovea (+) compared to the temporal fovea (*).

In the contralateral eye with severe NPDR (Fig. 3A), the enlarged FAZ is visible but the precise area is difficult to determine on FA (Fig. 3B,C). The FAZ on OMAG demonstrated a more extensive enlargement of the FAZ than what was expected from the fundus photos or FA without high magnification (Fig. 3E). Moreover, unlike FA, OMAG images were not disrupted by the blockage of signal from superficial retinal hemorrhages (Arrow in Fig. 3D).

Taken together, wide-field OCT imaging in this patient with diabetic retinopathy is superior to traditional FA images in both clarity and content. For future clinical studies, the size and content of neovascular complexes, no matter how minute, or blood flow velocity may prove to be important parameters in predicting diabetic retinopathy disease progression or response to treatment. The utility and significance of these parameters and their impact on diagnosis and treatment will need to be assessed in future studies. In the more immediate clinical setting, the greatest utility of OMAG may be in patients where the presence of neovascular complexes or other vascular pathology is equivocal based on clinical exam. In these cases, clinicians may more readily opt for a non-invasive OMAG study and treat accordingly as opposed to ordering an invasive FA study. In the latter scenario, clinicians may defer imaging altogether and monitor the area in question at future visits, thereby possibly delaying treatment. In addition to the higher information content provided by OMAG wide-field imaging, the ease of utility and non-invasive nature is likely to impact the diagnosis and management of retinal disease.

### Inner Retina: Branch Retinal Vein Occlusion

**Figure 4:**
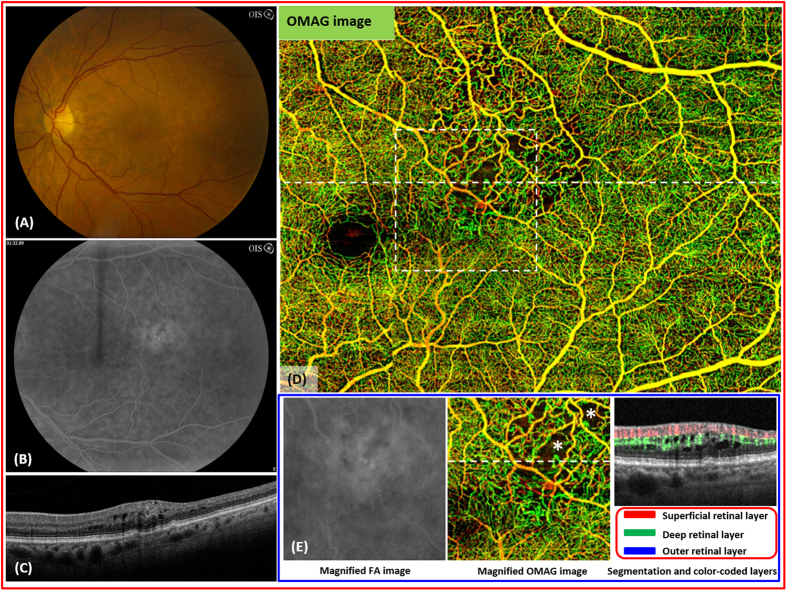
Imaging of branch retinal vein occlusion (BRVO) in the left eye of a 68 year-old female. (**A**), the fundus photo of the left eye shows several hemorrhages and edema in the superotemporal macula. (**B**), the arteriovenous phase of the fluorescein angiography shows leakage in the area of the BRVO. (**C**), the optical coherence tomography shows intraretinal fluid. (**D**), the whole retinal layer optical OMAG image illustrates a large area of capillary dropout and vascular irregularity in the superotemporal macula. (**E**), the flow image shows the evidence of interrupted flow within the deep retina layer associated with cystoid macular edema (*).

The branch retinal vein occlusion is a relatively common cause of visual loss^39^. The visual prognosis depends on the intact foveal capillaries as seen by fluorescein angiography^40^,^41^. However, the precise determination of the FAZ or the extent of macular capillary damage can be difficult with FA alone (Fig. 4B). A 68 year-old female with a BRVO of the left eye (Fig. 4A) has been imaged using a 3 × 4 single cube scan protocol. OMAG demonstrated capillary loss within the affected area with high-resolution detail (Fig. 4D,E). The area of vascular abnormalities was more extensive than what was expected from the FA leakage (Fig. 4D). Disrupted flow due to capillary dropout within the deep retinal layer (e.g. areas marked stars) and the resulting cystoid macular edema are detectable on cross sectional view (Fig. 4E). The degree of capillary nonperfusion is detectable in real-time. Information on the extent of vascular disruption and alterations in capillary flow may prove to be significant characteristics in determining prognosis and treatment of vein occlusions.

### Outer-retina, retinal pigment epithelium, choriocapillaries: Retinitis Pigmentosa

Retinitis pigmentosa is characterized by the progressive loss of rod photoreceptors and the retinal pigment epithelial layer. Loss of normal retinal vasculature is thought to be due to photoreceptor degeneration^33^. The progression of RP as evidenced by visual field tests is usually followed by changes on structural OCT. However, the extent of damage in retinal and choroidal vasculature is not detectable by traditional imaging modalities.

**Figure 5:**
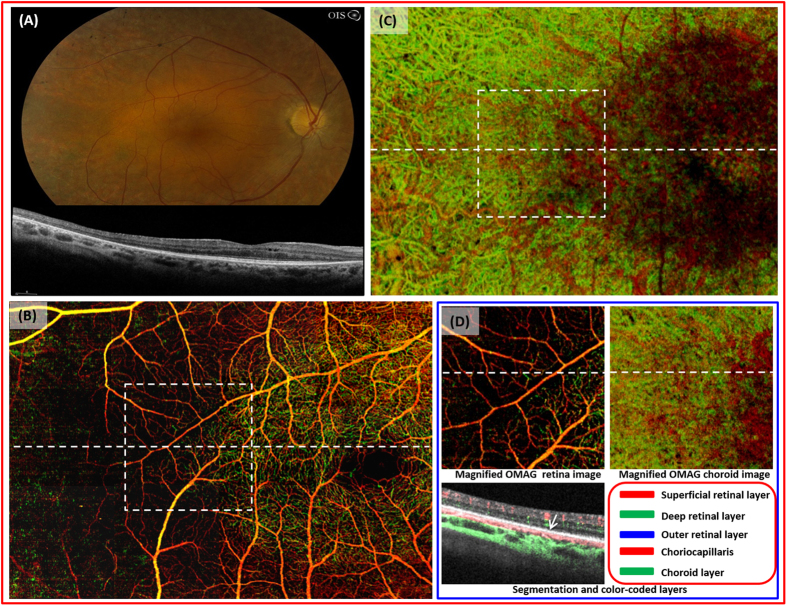
Imaging of a 43 year-old female with retinitis pigmentosa. (**A**), the fundus photo of the right eye accompanied with spectral domain OCT shows the atrophy of the outer retina and pigmentary changes in the mid periphery. (**B**), the OMAG image of the whole retinal layer demonstrates the absence of normal vasculature within the superficial and deeper retinal layer outside the central macula. The box indicates the magnified area shown in (**D**). (**C**), the choroid slab delineates the area where the outer retina and retinal vasculature integrity is intact. The box indicates the magnified area shown in (**D**). (**D**), the flow image of the area indicated by the dashed line of the retinal and choroid OMAG images shows relative absence of vascular flow within the deep retinal layer where there is outer retinal loss. (The outer retina is intact to the right of the arrow).

A 43 year-old female with RP has been imaged using a 3 × 4 single cube scan protocol. The fundus photo demonstrates attenuated vessels, optic nerve pallor, and pigmentary changes encroaching the central fovea (Fig. 5A). The loss of outer retina is detected on SD-OCT (Fig. 5A). OMAG imaging delineates the absence of superficial and deep retinal capillary networks (Fig. 5B). The signal intensity of the central macula in the choroid image is lower than the peripheral macula due to intact retinal vasculature and outer retina (Fig. 5C). Thus, the extent of the photoreceptor loss is visible at one glance. The segmented cross-sectional flow image demonstrates near absence of deep retinal layer blood flow where there is outer retinal loss (left to the arrow, Fig. 5D).

The loss of normal retinal vasculature is easily detectable with OMAG. In addition, the vascular flow within main retinal layers can be determined beyond the central macula. Therefore, OMAG may provide a useful tool to follow the progression of RP pathology in quantifiable vasculature loss and measure the degree of functional loss in patients. The loss of retinal vasculature may precede patients’ visual field loss and the integrity of retinal vasculature could become a new clinical endpoint. In addition, the ability to quantify and assess the retinal blood vessels may become more important in future with advances in stem cell or gene therapy^42^.

### Choriocapillaries, Choroid: Polypoidal Choroidal Vasculopathy

Initially described as an ill-defined, recurrent, subretinal hemorrhagic disorder^43^,^44^, polypoidal choroidal vasculopathy is a disease of the inner choroidal vasculature. ICG and OCT are currently the key imaging modalities in evaluating PCV but are limited in visualizing polyps. Recent efforts have focused in improving the sensitivity of choroidal vasculature detection with deeper penetration^45^.

**Figure 6:**
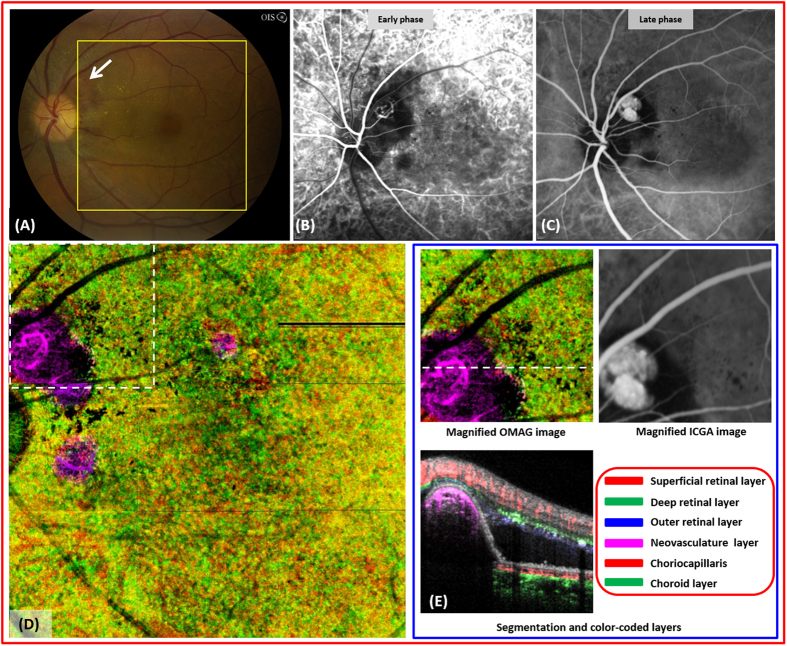
Imaging of polypoidal choroidal vasculopathy (PCV) in a 50 year-old female. (**A**), fundus photograph of the left eye shows a peripapillary, orangish mass under the retina (arrow). (**B,C**), the early and late frames of the indocyanine green angiography (ICG) demonstrates an active polyp. (**D**), the choriocapillaries and choroid slab shows a well-defined polyp located above choriocapillaries. (**E**), magnified OMAG and ICG image of the area marked by dashed white box in (**D**). The flow image shows blood flow within the active polyp.

A 50 year-old female with PCV of the left eye was imaged using a 3 × 3 single cube scan protocol. An active polyp in the peripapillary region demonstrates leakage on ICG (Fig. 6B,C). The subretinal, orange mass on the fundus photo (Fig. 6A, arrow) is easily detected as a vascular lesion on the choroid OMAG imaging (Fig. 6D). The color of the polyp is different from the rest of the choroid due to its elevation. The segmentation flow image is complementary to the enface image in demonstrating a large, elevated, choroidal mass (Fig. 6E). Strong signal intensity within the polyp suggests brisk flow and activity.

**Figure 7:**
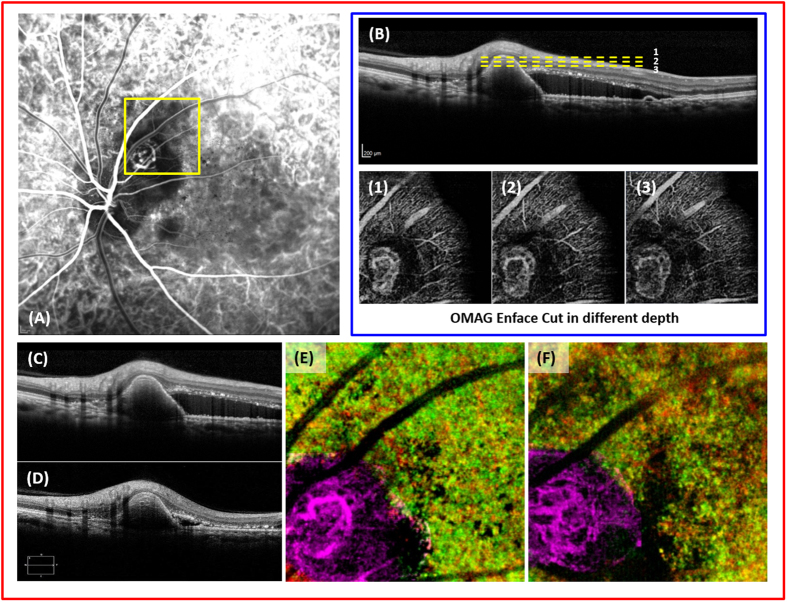
Imaging of polypoidal choroidal vasculopathy (PCV) before and after treatment with anti-vascular endothelial growth factor (anti-VEGF). (**A**), the indocyanine green angiography of the left eye. (**B**), cross-sectional image of the polyp and en face images of the polyp dissected at three different levels (yellow, dashed lines) (**C**), the optical coherence tomography image of the polyp before treatment. (**D**), the optical coherence tomography image of the polyp and reduction of the subretinal fluid after treatment with anti-VEGF. (**E,F**), enface images of the polypoidal lesion before and after treatment with anti-VEGF.

The structural OMAG image from before and after treatment with anti-vascular endothelial growth factor shows a remarkable reduction in subretinal fluid (Fig. 7C,D). However, the size and shape of the polyp remained relatively stable (Fig. 7E,F). Given that the vasculature change within the polyp is easily appreciable, further follow up studies with OMAG imaging may lead to quantifiable outcome measures, which are currently lacking in the evaluation of PCV (e.g. Fig. 7A,B).

## Discussion

The advent of the OCT angiography has enabled us to differentiate various retinal pathologies of the microvasculature at all retinal and choroidal levels. This study has summarized the results of the OMAG imaging with the aid of LSO based motion-tracking system. Its remarkable reproducibility and high signal to noise ratio allowed several image cubes scanned from different locations to be montaged automatically, enabling unprecedented wide-field imaging with high definition.

There have been several methods of correcting the motion-artifacts in OCT angiography, such as intensity-based correlation approach^46^ or Doppler OCT phase based motion compensation^47^. Additionally, more complex approaches of combining both intensity and phase based-algorithms have been described^48^. The system described here is equipped with motion tracking through an auxiliary real time line scan ophthalmoscope that enabled ultra-high definition retinal and choroidal vasculature imaging *in vivo*. In comparison to FA or ICG, the OMAG images were able to detect not only the presence of retinal/choroidal vascular abnormalities but also their exact spatial location within the retinal layers, the caliber of abnormal vessels, the exact size of neovascular complexes, and the extent of damaged normal vasculature. The ability to detect these information non-invasively will likely make OMAG a highly informative tool for diagnostic and follow up studies.

There are several limitations of OMAG. The image quality is somewhat dependent on patient cooperation, and processing of high-resolution images can be time consuming. It is important to note that there is no standard way of evaluating OCT angiography currently and new clinical endpoints need to be established with larger studies. Like all the other OCTA algorithms, another limitation for OMAG is the well-known projection artifacts due to superficial blood flows that give rise to a difficulty in interpreting the blood vessel networks within deep retinal layers. The projection artifacts are the main cause of yellow color appearance in the false color OMAG images as shown in the result section. Although there are several methods proposed to minimize projection artifacts in OCTA images of choroid^49^,^50^, we did not attempt to use these methods in the presentation of the current results of deep retinal layers because they have not been validated for the retinal layers. Future work is needed to develop the projection artifact removal algorithm that is applicable for the retinal layers.

Nevertheless, we are approaching the era that newer imaging modalities are delivering higher definition and deeper penetration images than ever possible before. As a result, objective measures in previously descriptive features of many diseases are likely to be established in future and incorporated in disease classification and treatment. The quantification of the FAZ or ischemic area in diabetes or vein occlusion, the determination of the size, volume, and shape of polyps in PCV, or the measurement of vasculature loss in RP are only a few, exciting examples of new metrics for retinal diseases.

Our study results show that the ability to perform LSO based motion-tracking is a key advance in the field that allows wider field of view images with high definition. OMAG would be a powerful tool in functional imaging of the retinal and choroidal vasculature. Clinical applications and OCTA based new, clinical endpoints should be validated in larger studies.

## Additional Information

**How to cite this article**: Zhang, Q. *et al.* Wide-field optical coherence tomography based microangiography for retinal imaging. *Sci. Rep.* **6**, 22017; doi: 10.1038/srep22017 (2016).

## Supplementary Material

Supplementary Information
