# Supplementary material for: Wide-field optical coherence tomography based microangiography for retinal imaging
Source: Sci Rep. 2016 Feb 25;6:22017. doi: 10.1038/srep22017 (PMC4766473; doi:10.1038/srep22017)
Supplement: Supplementary Information [file srep22017-s1.pdf]

Wide-field optical coherence tomography based microangiography for retinal imaging

Qinqin Zhang[1]†, Cecilia S. Lee[2]†, Jennifer Chao[2], Chieh-Li Chen[1], Thomas Zhang[3], Utkarsh Sharma[3], Anqi Zhang[1], Jin Liu[1], Kasra Rezaei[2], Kathryn L Pepple[2], Richard Munsen[2], James Kinyoun[2], Murray Johnstone[2], Russell N Van Gelder[2], and Ruikang K Wang[1, 2]*

† *These authors have contributed equally to the manuscript and should be considered equivalent first authors.*
* Corresponding author

1. University of Washington, Department of Bioengineering, 3720 15th Ave NE, Seattle, WA 98195, USA
2. University of Washington, Department of Ophthalmology, 325 Ninth Avenue, Seattle, WA 98104, USA
3. Carl Zeiss Meditec, Inc., Dublin, CA 94568, USA

<u>Supplementary Figures</u>

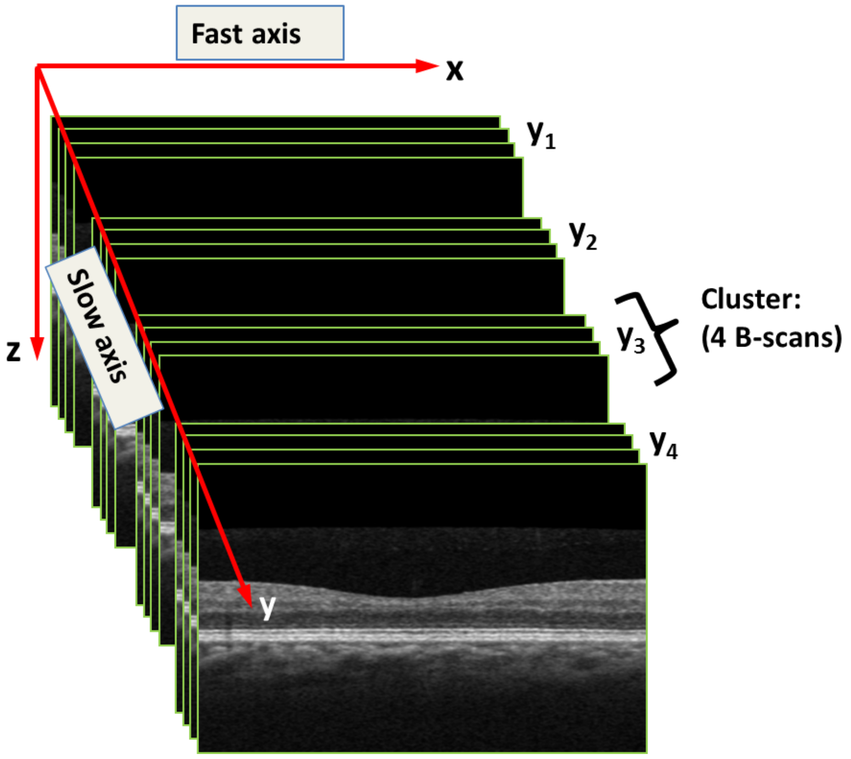

Supplementary Figure 1. The scanning protocol for OCT angiography used in the current study for each cube scan. “x” represents the fast scan axis (i.e., B-scan direction) and “y” indicates the slow scan axis, where at each y scan location, a cluster of repeated B-scans (4 times in the current study) are performed to extract the flow signal.

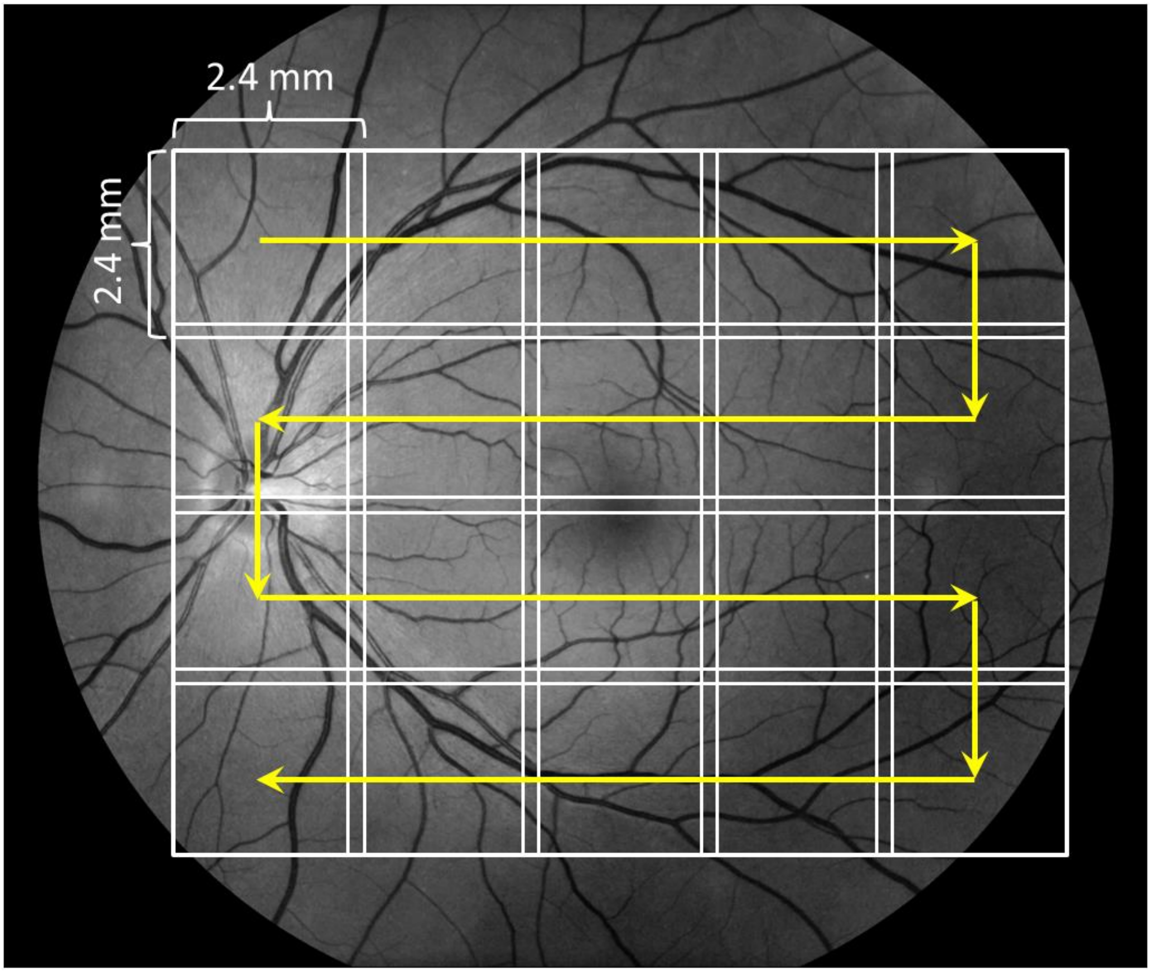

Supplementary Figure 2. Montage-scanning protocol: The grid pattern (or coordinates of each cube) is pre-defined by the user depending on the size requirement of coverage area in the posterior segment of the eye. With the help of line scanning laser ophthalmoscope, the system automatically scans the retina one cube after another, starting from the top left corner. Arrowed yellow line indicates the order of cubes being scanned. Each single cube is of 2.4 x 2.4 mm2 in size on the retina. Adjacent cubes have 10% overlap in order for later montaging to form a wide-field image of 3D OMAG and OCT.
